# On the Network Convergence Process in RPL over IEEE 802.15.4 Multihop Networks: Improvement and Trade-Offs

**DOI:** 10.3390/s140711993

**Published:** 2014-07-07

**Authors:** Hamidreza Kermajani, Carles Gomez

**Affiliations:** 1 Universitat Politècnica de Catalunya/Fundació i2Cat, C/Esteve Terradas, 7, 08860 Castelldefels, Spain; E-Mail: carlesgo@entel.upc.edu; 2 Department of Computer Engineering, Toyserkan Branch, Islamic Azad University, Toyserakn, Hamedan Province, 65816-85184, Iran

**Keywords:** RPL, IPv6, IEEE 802.15.4, network convergence, Trickle, RPL parameters

## Abstract

The IPv6 Routing Protocol for Low-power and Lossy Networks (RPL) has been recently developed by the Internet Engineering Task Force (IETF). Given its crucial role in enabling the Internet of Things, a significant amount of research effort has already been devoted to RPL. However, the RPL network convergence process has not yet been investigated in detail. In this paper we study the influence of the main RPL parameters and mechanisms on the network convergence process of this protocol in IEEE 802.15.4 multihop networks. We also propose and evaluate a mechanism that leverages an option available in RPL for accelerating the network convergence process. We carry out extensive simulations for a wide range of conditions, considering different network scenarios in terms of size and density. Results show that network convergence performance depends dramatically on the use and adequate configuration of key RPL parameters and mechanisms. The findings and contributions of this work provide a RPL configuration guideline for network convergence performance tuning, as well as a characterization of the related performance trade-offs.

## 1. Introduction

Wireless Sensor Networks (WSNs) are experiencing significant momentum in academic, industrial and standardization circles. WSNs comprise sensor and actuator nodes that enable intelligent monitoring and control applications in a wide spectrum of environments including smart cities, home automation, remote health and precision agriculture, to mention but a few [1].

A plethora of communication protocols for WSNs have emerged [2]. Among these, the contribution of the IETF is of outstanding importance, since this organization is producing IP-based protocol specifications that allow the connection of WSNs to the Internet, and thus enable the Internet of Things (IoT) [3]. Because many WSNs are multihop, and thus require a routing protocol, the IETF Routing over Low-Power and Lossy Networks (ROLL) working group [4] recently developed the IPv6 Routing Protocol for Low-power and Lossy Networks (RPL) [5]. RPL was specifically designed to meet the requirements of WSNs [6–9] and is a central component of the IETF protocol suite for the IoT. Therefore, since RPL is expected to be widely deployed, it is fundamental to characterize its properties, and evaluate the influence of its main parameters and options on network performance.

Despite its novelty, RPL has already been a subject of study [10–21]. Most of the literature focuses mainly on evaluating RPL behavior in steady state [16–21]. However, performance of RPL during network convergence may be critical, since it may significantly affect user experience (e.g., when a user expects fast network creation to fulfill a certain action) and it is fundamental to global network recovery due to topology changes. Nevertheless, RPL network convergence has received limited attention. The studies that consider RPL performance in transient state do not provide a deep analysis, since they do not focus on the joint influence of RPL parameters and mechanisms, and network characteristics (such as network size and density), on network convergence performance [10–17,21].

In this paper we investigate by simulation the influence of the main RPL parameters on the network convergence process, and we also propose and evaluate a mechanism that leverages an option available in RPL for spurring the network convergence process. The performance parameters we consider are network convergence time, network join time and message overhead. In order to assist the derivation of conclusions, we also evaluate the number of collisions during network convergence. With the aim of obtaining comprehensive results, we carry out extensive simulations for a wide range of conditions in terms of network size and density. We consider multihop networks whereby the nodes use IEEE 802.15.4, *i.e.*, the most prevalent radio interface for WSNs. In order to achieve realistic results, we have tuned the simulation environment in order to accurately model the link behavior observed in real experiments. However, the results presented should be confirmed on a real testbed in a future work.

Results show that RPL network convergence performance depends dramatically on the use and adequate configuration of key parameters and mechanisms. The findings and contributions of this work provide a guideline for configuring and selecting adequately crucial RPL parameters and mechanisms for achieving high network convergence performance, on the basis of network characteristics such as size and density, as well as a characterization of the related performance trade-offs.

The remainder of this paper is organized as follows: Section 2 reviews the main characteristics of IEEE 802.15.4. Section 3 gives an overview of RPL, with special focus on the procedures for network creation. Section 4 describes the simulation environment and methodology used in the paper. Section 5 studies the impact of the main RPL parameters on performance of the network convergence process. Section 6 evaluates a mechanism proposed by the authors for accelerating network convergence, leveraging an option available in RPL. Section 7 reviews related work and, finally, Section 8 presents the main conclusions of the paper.

## 2. IEEE 802.15.4 Main Characteristics

This section gives a short summary of the main IEEE 802.15.4 characteristics. IEEE 802.15.4 is the de-facto family of standard radio interfaces for low-cost, low-power, and low-rate wireless communications. IEEE 802.15.4 specifies Physical (PHY) and Medium Access Control (MAC) layer functionality [22].

The widely implemented IEEE 802.15.4-2003 specification defines three physical layers in the 868 MHz, the 915 MHz and the 2.4 GHz bands, which offer raw data rates of 20 kbit/s, 40 kbit/s and 250 kbit/s, respectively. The latter physical layer, which uses an Industrial, Scientific and Medical (ISM) band available worldwide, is the most commonly used one.

At the MAC layer, IEEE 802.15.4 networks may be configured in two modes: (i) on the basis of a superframe structure delimited by beacons, or (ii) in a beaconless mode, which is generally preferred due to its simplicity, whereby nodes use unslotted CSMA/CA for medium access. In this mechanism, the process for transmitting a data frame requires the sender to wait for an initial random backoff time. Subsequently, the sender performs Clear Channel Assessment (CCA) to check the state of the medium. If the medium is idle, the sender carries out the transmission. Otherwise, the sender performs a new backoff, of a duration randomly chosen from a greater window of possible values, and evaluates the medium state again. This procedure is repeated several times if the medium is always found to be busy. If the number of unsuccessful channel access attempts reaches a maximum limit, the frame transmission fails and the frame is discarded.

IEEE 802.15.4 offers optional reliability for unicast frames by allowing the request of acknowledgment frames. Broadcast frames are not acknowledged. Network layer multicast packets (see subsection 3.1) are encapsulated in broadcast frames in IEEE 802.15.4.

## 3. RPL Overview

In this section we provide a RPL overview, with emphasis on the procedures for network creation. The section is organized in three parts. First, we present the mechanisms used by RPL to build a network. We then briefly review the routing mechanisms in RPL. Finally, we present the Trickle algorithm, which is used by RPL to disseminate control messages for network creation and maintenance.

### 3.1. DODAG Construction

RPL builds and maintains network structures called Destination Oriented Directed Acyclic Graphs (DODAGs). A DODAG is defined as a graph composed of nodes and links that constitute paths that point towards and terminate at a special node called the root. The DODAG root represents a node that generally performs the tasks of a sink or a gateway. RPL has been designed and optimized for the transmission of data from sensor nodes towards the root node. In theory, cycles do not exist in a DODAG, which inherently provides protection against the problem of loops. However, researchers have shown that there may exist transitory loops in RPL DODAGs [23,24].

For the construction and maintenance of a DODAG, RPL nodes locally multicast DODAG Information Object (DIO) messages pseudo-periodically, by using the Trickle algorithm [25] (see Section 3.3). A DIO message contains information that allows a node to discover an existing DODAG, learn node and network configuration parameters, and maintain the network topology.

In order to join a DODAG, a node must receive at least one DIO message from a neighbor. To this end, the node may either wait to receive DIO messages from its neighbors or may multicast a DODAG Information Solicitation (DIS) message to request the immediate transmission of DIO messages from neighboring nodes. When a DODAG node receives a DIS message, it schedules itself to send out quickly a DIO message. Once a node has sent a DIS message, it may send further DIS messages until the node receives a DIO message in response. If no DIO message is received after a given period of time, the node may decide to become the root of a new DODAG.

Each node in a DODAG has a rank, which abstracts the topological distance between a node and the DODAG root. Each DODAG node indicates its rank in the DIO messages it transmits. A node calculates its rank by applying the Objective Function (OF) in use in the DODAG, and using the rank of its neighbors as an input. The OF defines how nodes use one or more metrics and constraints in order to determine their own rank. Any RPL implementation must support OF Zero (OF0), a metric-agnostic OF that allows interoperability between implementations in a wide spectrum of use cases [26].

**Figure 1.**
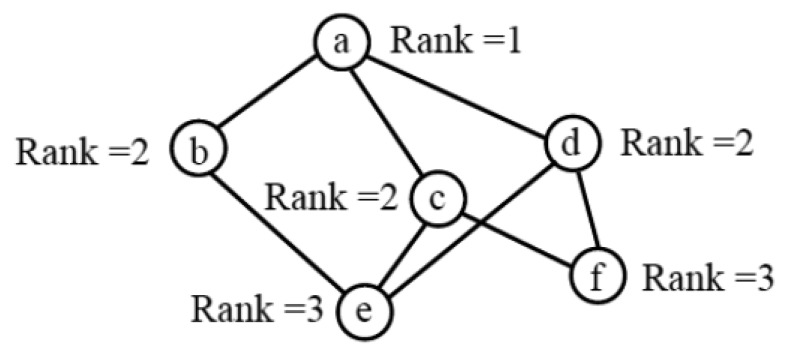
An example DODAG composed of 6 nodes, where node a is the DODAG root.

Based on the DIO messages received, each DODAG node chooses the members of its DODAG parent set, which is a subset of the DODAG node neighbors that provide a path towards the root. All members of the parent set of a node must have a lower rank than the node. For example, in Figure 1, the parent sets of nodes *e* and *f* are {*b,c,d*} and {*c,d*}, respectively.

### 3.2. Routing in RPL

RPL provides basically two types of routes, which depend on the endpoints involved in the communication: upward and downward routes. The former offer a path to the DODAG root from any other node. A downward route provides a path in the opposite direction.

As described in the previous subsection, after joining a DODAG, each node chooses its parent set. Every node selects its preferred parent among the members of its parent set by applying the OF in use in the DODAG. The preferred parent of a node offers the default upward route for this node. RPL supports downward routes by using Destination Advertisement Object (DAO) messages. DAO messages are generated and sent upwards by non-root nodes to announce themselves as possible destination nodes. In order to enable Point-to-Point (P2P) flows, *i.e.*, the transmission of data between any two network nodes, RPL offers using upward and downward routes as the default mechanism. Since this mechanism may not be optimal, an improved mechanism for P2P traffic called P2P-RPL has been developed [27]. P2P-RPL allows a node to discover a P2P route on demand, by creating a temporary DODAG rooted at the node.

### 3.3. Trickle Algorithm

As mentioned in Section 3.1, RPL uses the Trickle algorithm to schedule and transmit DIO messages in a network [25]. Therefore, Trickle plays a key role in the convergence of a RPL network. Trickle is a density-aware local multicast communication mechanism that is based on a consistency model. According to this model, a network is in a consistent state when it does not suffer changes and therefore DIO messages are consistent with prior DIO messages transmitted. In this case, nodes slow their DIO message rate exponentially. However, when a node detects an inconsistency, it increases its DIO message transmission rate to resolve the inconsistency quickly. On the other hand, Trickle supports a message suppression mechanism that aims at minimizing the DIO message redundancy in a network. A detailed presentation of the Trickle algorithm is provided next.

Trickle divides time into non-identical intervals. In each interval, every node attempts to send one DIO message. Trickle rules determine whether the transmission will be allowed or not. The Trickle algorithm operates based on a set of parameters, variables and rules, which are reviewed next.

There are three parameters that configure the Trickle algorithm:

- The minimum interval size, *I_min_*.
- The maximum interval size, *I_max_*.
- The redundancy constant, *k*.

In addition, the Trickle algorithm uses three variables that track the current status of the algorithm. These variables are: the current interval size, *I*, the tentative transmission time within the current interval, *t*, and the number of messages heard within the current interval, *c*.

The operation of Trickle is based on the following steps:

1. Initially, Trickle sets *I* to a value in the range [*I_min_,I_max_*].
2. At the beginning of each interval, Trickle resets *c* to 0 and sets *t* to a random point within the range [*I*/2,*I*).
3. Whenever Trickle hears a transmission that is consistent, it increments its own counter *c*.
4. At time *t*, Trickle performs the transmission if, and only if, its counter *c* is smaller than the redundancy constant, *k*; otherwise, the transmission is suppressed.
5. Whenever the interval *I* expires, Trickle doubles the interval length (if the new interval length is greater than *I_max_*, the new interval length is set to *I_max_*). Then, the algorithm goes back to execute step 2.
6. If Trickle hears a transmission that is inconsistent, it must reset *I*, set this variable to *I_min_*, start a new interval and execute step 2.

The three main parameters of the Trickle algorithm, *I_min_, I_max_* and *k*, are set by the DODAG root. The values for these parameters are encapsulated in the DIO messages sent by the root. Hence, these parameter values are learnt by the root neighbors upon DIO reception, and are included in the DIO messages sent by these neighbors. The same process happens for the rest of nodes that join the DODAG, and thus the parameter values originally announced by the root are propagated to the whole DODAG. In order to keep a low network convergence time, *I_min_* is commonly used as the value for the first interval [28].

## 4. Simulation Environment and Methodology

This section presents the simulation environment and methodology used to evaluate the network convergence process of RPL in IEEE 802.15.4 multihop networks.

In order to carry out our simulations, we used OMNeT++ [29], a well-known C++-based discrete event simulator, jointly with MiXiM, an OMNeT++ framework created for various types of wireless networks [30]. We implemented RPL for this simulation environment. As a side contribution, we have made the simulation code publicly available [31]. The simulated network nodes were static and located following a uniformly random spatial distribution in two-dimensional square areas. We configured the nodes to use the 2.4 GHz band IEEE 802.15.4 physical layer, and the beaconless mode functionality. We set the MAC queue length of the nodes to 1 in order to replicate the value of this parameter in CC2420, a widely used IEEE 802.15.4 radio chip [32]. We used the log-normal shadowing propagation model available in MiXiM. In order to achieve accurate and realistic link behavior in our simulation framework, we carried out experiments and tuned the parameter settings of the simulated propagation model based on the experimental results. The experiments comprised communication in a link between two TelosB nodes [33] running TinyOS [34]. Table 1 shows the main physical and link layer parameter settings used in the simulations.

**Figure 2.**
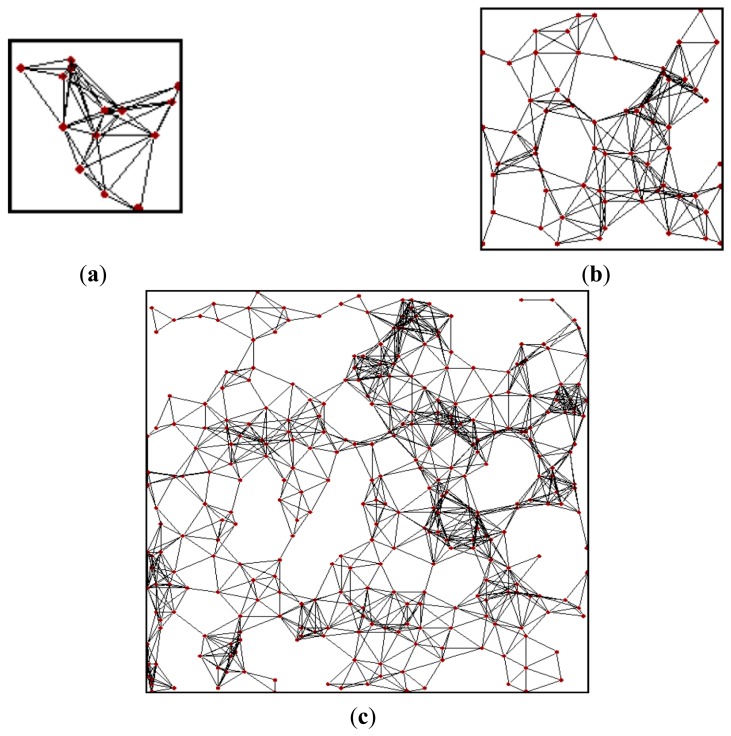
Illustration of network nodes and existing links in example instances of (**a**) small, (**b**) medium, and (**c**) large scenarios, for an average node degree of 10. The depicted scenarios have been plotted to scale.

In order to evaluate the influence of the network size in our study, we considered 3 different scenarios, denoted small, medium and large network scenarios. The area of the large network scenario is 100 × 100 m^2^, which is 5 times greater than the medium network area. There is the same relationship between the medium and small network scenario areas. Figure 2 depicts the nodes' location and existing links in example instances of small, medium and large network scenarios, for a node degree of 10. We also considered the network density in our study, by evaluating node degree values of 5, 10 and 15. Note that this range of network densities covers from relatively sparse networks to highly connected and dense networks. As a result, we simulated a variety of network sizes and densities, ranging from 8-node to 483-node networks, which cover a wide range of use cases. The number of nodes and the average rank for each network size and density scenario are shown in Table 2.

**Figure 3.**
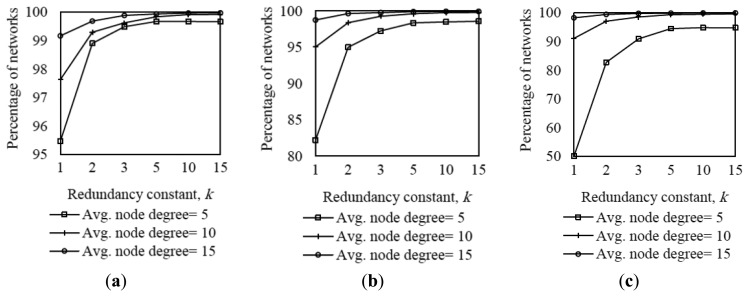
Percentage of networks converged in less than 10,000 s in three network size scenarios, for different node densities, as a function of the redundancy constant, *k*: (**a**) Small network scenario, (**b**) medium network scenario, and (**c**) large network scenario, respectively.

For each individual combination of network size scenario, network density and protocol configuration, we simulated 1500 different randomly generated topologies, and evaluated 20 instances of the network convergence process for each topology, yielding a total of 30,000 network convergence instances. We discarded the results of DODAGs not formed in less than 10,000 s. We applied this measure since we observed that, in certain scenarios, the time until all nodes receive at least one DIO message may be significantly longer than 10,000 s, whereas network convergence requirements for most deployments are 3–5 orders of magnitude below that threshold [6–8]. In order to characterize this phenomenon, we evaluated the percentage of networks formed in less than 10,000 s in the scenarios considered in subsection 5.1: small, medium and large network scenarios, for different node density and *k* values, when *I_min_* is set to the default value, *i.e.*, 8 ms (see Figure 3). In the described conditions, the minimum and maximum percentage of times that networks formed in less than 10,000 s are 50.14% and 99.97%, which correspond to the large network scenario when the node degree is 5 and *k* is 1, and the small network scenario when the node degree and *k* are 15, respectively. As shown in Figure 3, the percentage of networks not formed in less than 10,000 s grows as the network density decreases (since nodes have a lower amount of neighbors from which DIO messages can be received), as the redundancy constant decreases (because a greater number of DIO message transmissions are suppressed), and as the network size increases (since the average number of end-to-end hops between the root and the rest of nodes increases as well).

## 5. Evaluation: Influence of the Main RPL Parameters on Network Convergence Performance

In this section we study the influence of the two most crucial RPL parameters on the performance of the network convergence process, in the network scenarios presented in the previous section. The two considered RPL parameters are the redundancy constant, *k*, and the minimum interval, *I_min_*. This section comprises two subsections, which focus respectively on the impact of each one of the aforementioned RPL parameters on network convergence performance.

### 5.1. Influence of the Redundancy Constant on the Network Convergence Process

One of the RPL parameters that affects network convergence performance to a greatest extent is the redundancy constant, *k*. As presented in subsection 3.3, this parameter limits the number of transmitted messages per Trickle interval in a given coverage area. In order to analyze the influence of the redundancy constant on the network convergence process, we simulated the network size and density scenarios shown in Table 2 for a range of redundancy constant values between 1 and 15, which includes the default value for this parameter stated by the RPL specification (*i.e., k* = 10) [5]. For the results presented in this subsection, the rest of RPL parameters were set to the default values stated in the RPL specification (note that *I_min_* was therefore set to 8 ms). We evaluated network convergence time, DODAG join time of a node, number of DIO messages transmitted, and number of collisions.

**Figure 4.**
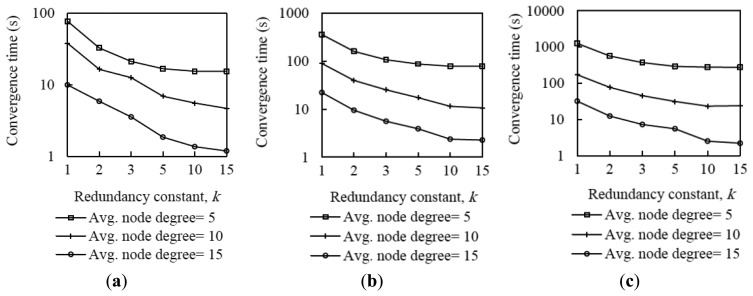
Average network convergence time in three network size scenarios, for different densities, as a function of the redundancy constant, *k*: (**a**) Small network scenario, (**b**) medium network scenario, and (**c**) large network scenario, respectively.

Figure 4 shows average network convergence time in small, medium and large network scenarios, for different node density and *k* values. To obtain the convergence time of a network, we calculated the time from the instant in which the DODAG root starts its first Trickle interval until the instant in which all nodes have joined the DODAG. We consider that a non-root node joins a DODAG when it receives a DIO message for the first time.

As it can be seen in Figure 4, using low values for the redundancy constant yields high network convergence time in all network size and density scenarios considered. This behavior is mainly due to the message suppression mechanism in the Trickle algorithm. When the root node transmits its first DIO message, the neighbors of the root that receive this DIO message schedule themselves to send their own first DIO message. However, when *k* is set to a low value, only a few neighbors of the root will be allowed to transmit their DIO message in the current interval. If the rest of root neighbors (if any) hear a number of DIO messages greater than or equal to *k* in the current interval, they will suppress their DIO message transmission. As a result, nodes that are only neighbors of the latter root neighbors have to wait for subsequent intervals to have the opportunity to receive their first DIO message. The same phenomenon happens as DIO messages propagate through the network, which finally leads to high network convergence time.

As the redundancy constant increases up to medium values, the network convergence time decreases. This occurs because, with greater redundancy constant values, the probability that nodes suppress their DIO messages is lower. Therefore, the probability that a node receives its first DIO message earlier is greater. However, network convergence times do not vary significantly as the value of *k* increases beyond the values around the node degree. The reason for this behavior is that for such values of *k*, the number of DIO message suppressions is low, since many nodes have a number of neighbors lower than *k*, and thus varying *k* within the mentioned range does not significantly affect the network convergence time.

As is also shown in Figure 4, network convergence time decreases with the node degree since greater network density provides better network connectivity and a greater amount of opportunities for a node to receive DIO messages from its neighbors.

By analyzing Figure 4 results in deeper detail, we found that the relative impact of the redundancy constant on network convergence time does not vary significantly with the network size when networks are sparse. However, as the network density increases, the improvement that can be achieved by increasing the redundancy constant grows with the network size. For example, when the node degree is 15, in the small network size scenario, a network convergence time decrease factor of 8.3 can be achieved by using *k* = 15 in comparison with the result obtained for *k* = 1. However, for the same node density, the network convergence time decrease factor that can be achieved by comparing the use of *k* = 15 with *k* = 1 in the large network scenario is 14.5.

**Figure 5.**
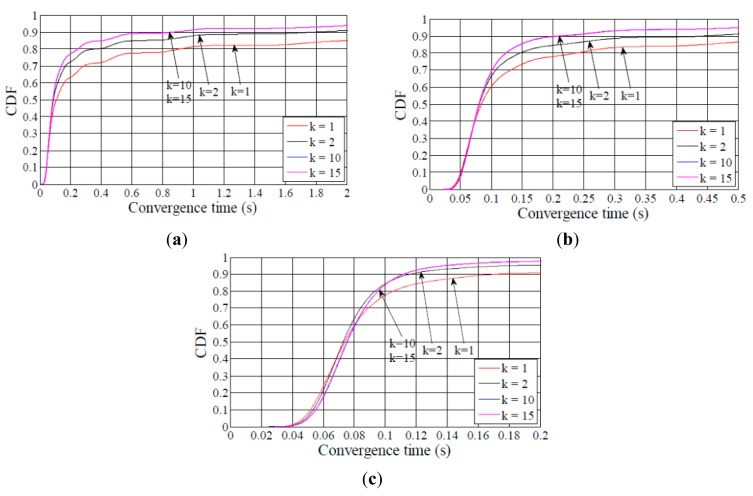
CDF of network convergence time in the small network size scenario. The average node degrees are 5, 10 and 15 in figures (**a**), (**b**) and (**c**), respectively.

**Figure 6.**
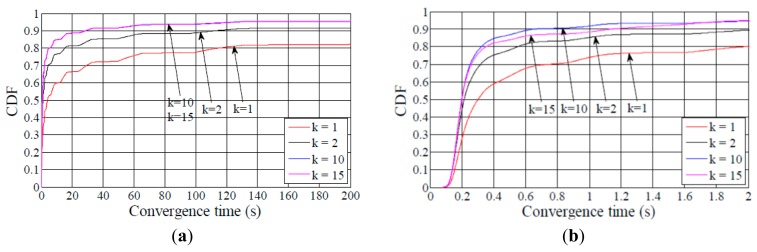

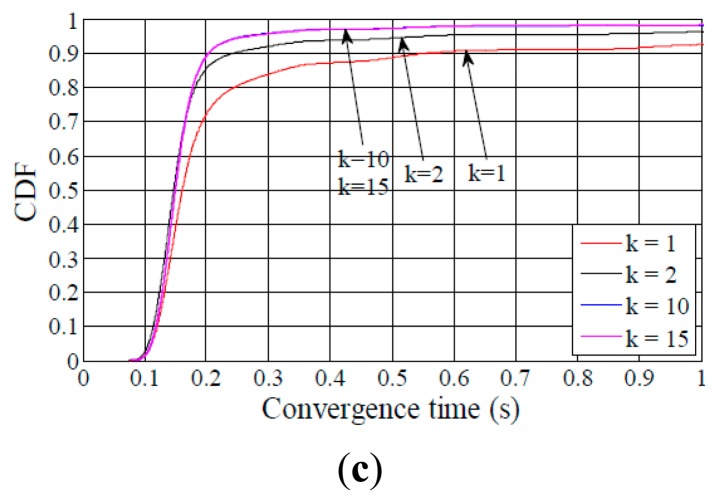
CDF of network convergence time in the medium network size scenario. The average node degrees are 5, 10 and 15 in figures (**a**), (**b**) and (**c**), respectively.

**Figure 7.**
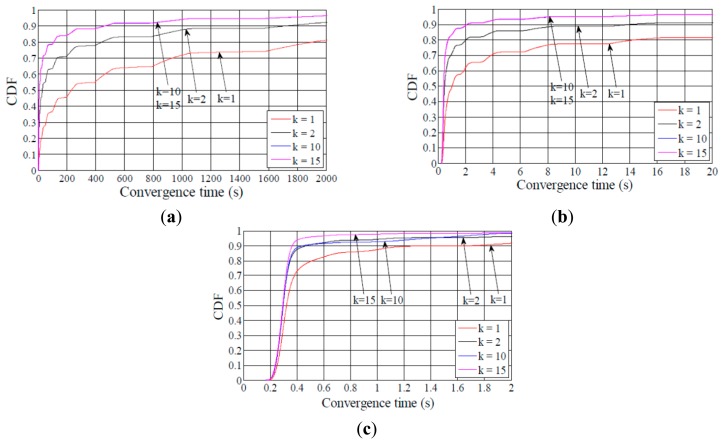
CDF of network convergence time in the large network size scenario. The average node degrees are 5, 10 and 15 in figures (**a**), (**b**) and (**c**), respectively.

Figures 5, 6 – 7 show the Cumulative Distribution Function (CDF) of the network convergence time for all the network size and density scenarios considered. The results illustrated by these figures evidence the dramatic impact of the redundancy constant on network convergence time. Increasing the redundancy constant value causes the percentage of DODAGs formed in a given time to increase as well. For example, in the medium network scenario, when the node degree is 5 (Figure 6a), and *k* is equal to 1, more than 80% of the DODAGs are formed in less than 120 s, while the same ratio of DODAGs are formed in less than 18 s for *k* ≥ 2. As the network density grows, network convergence times decrease as well. Still in the medium network scenario, when the node degree is 10 (Figure 6b), and *k* is set to 1, 80% of the DODAGs are formed in less than 2 s, whereas the same ratio of DODAGs are formed in less than 0.6 s for *k* ≥ 2. In the same scenario, for a node degree of 15 (Figure 6c), 90% of the DODAGs are formed for any value of *k* in less than 0.6 s.

On the other hand, the reader may observe the wave shape that can be appreciated in Figures 5, 6 and 7, especially when the node degree is low. This behavior is due to the interval duplication of the Trickle algorithm, and reflects the distribution of the number of intervals needed to completely form a DODAG, as well as the size of the interval in which a DODAG is formed.

**Figure 8.**
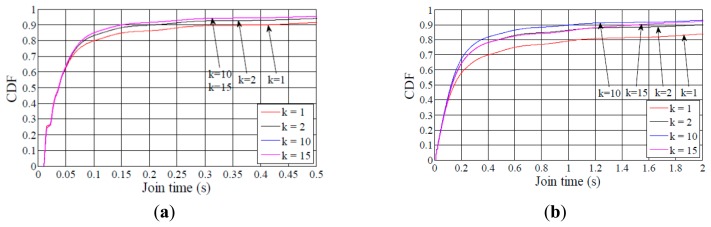

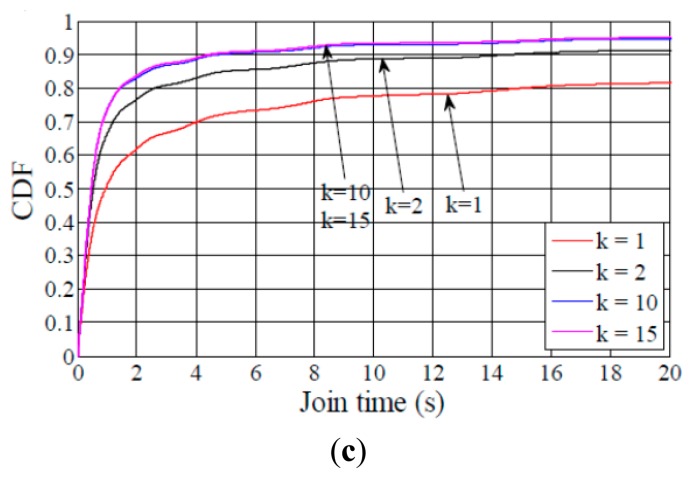
CDF of nodes join time for an average node degree of 5: (**a**) small, (**b**) medium, and (**c**) large network size scenarios.

Another useful performance result is the time it takes for a node to join a DODAG. Figure 8 shows the CDF of the DODAG join time for a node in the considered network scenarios. When the average node degree is 5 and *k* is equal to 1, around 80% of the nodes have joined the DODAG in 0.1 s, 1.1 s and 15 s in the small, medium and large network size scenarios, respectively. These times are significantly smaller than the average network convergence times shown in Figure 4. Therefore, a relatively small fraction of the nodes require a large amount of time to join a DODAG. Whether this is a critical aspect for the user depends on the application requirements the network is deployed for.

As shown in Figures 5, 6, 7 – 8, the difference between the curves for *k* = 1 and for *k* = 2 is greater than the difference between the curve for *k* = 2 and any curve for *k* > 2. This result illustrates that the network convergence time and join time improvement that can be achieved by increasing the redundancy constant decreases as the redundancy constant grows.

**Figure 9.**
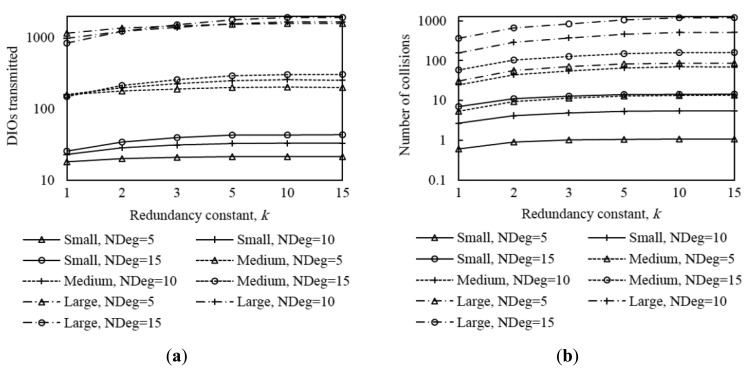
Average number of DIO messages transmitted (**a**) and collisions (**b**) in three network size scenarios, for different network densities, as a function of the redundancy constant, *k*. NDeg denotes the average node degree.

In order to provide insight on the influence of the redundancy constant on network convergence performance, we also evaluated the average number of DIO message transmissions and collisions that occur from the first DIO transmission by the root until the instant in which all nodes join the DODAG, for all the network size and density scenarios considered. The corresponding results are shown in Figure 9.

The average number of DIO message transmissions increases as the redundancy constant grows up to the node degree. In fact, increasing the redundancy constant within this range of values leads to a lower number of DIO message suppressions. When the redundancy constant is greater than the node degree, varying this RPL parameter does not have a significant effect on the number of DIO messages transmitted, since the number of message suppressions is very low. The number of collisions varies with *k* similarly to the number of DIO messages transmitted, since the number of DIO message collisions grows with the number of DIO messages that are actually transmitted. Note that despite the number of DIO message collisions increase with the redundancy constant, the network convergence time decreases with the redundancy constant. This happens because, as the redundancy constant grows, even though a subset of the additional DIO messages transmitted are lost due to collisions, a greater number of DIO messages are received correctly by non-DODAG nodes. Finally, we found that the relative influence of *k* on the number of DIO messages transmitted, and to a lower extent, on the number of collisions, increases with both network size and network density. As network size grows, the influence of *k* increases due to the multiplicative effect of a greater hop count between the root and any other node.

From the results shown in Figures 4, 5, 6, 7, 8 – 9, it can be concluded that there is a tradeoff between network convergence time (and node join time) and the number of DIO messages sent, which depends on the redundancy constant. In order to achieve a low network convergence time, the redundancy constant should be set to a value equal or close to the average node degree. Greater redundancy constant values might cause an unnecessary increase in the number of DIO messages transmitted and collisions in very dense areas of a network, which however would not yield a network convergence time decrease. The influence of the redundancy constant grows with both the network density and size.

### 5.2. Influence of the Minimum Interval on the Network Convergence Process

In this subsection we study the effect of the minimum interval, *I_min_*, on the network convergence process. We evaluate the impact of *I_min_* on the network convergence time, number of DIO message transmissions and number of collisions during the network convergence process. We also analyze the relationship among these performance parameters. The RPL specification states that the default value for *I_min_* is 8 ms [5]. However, in order to investigate the influence of *I_min_* on network convergence performance, we evaluate different *I_min_* values in the same scenarios and for the same redundancy constant values considered in the previous subsection.

**Figure 10.**
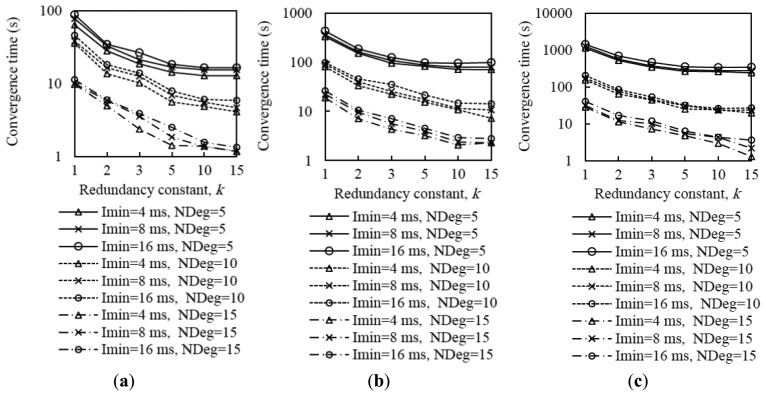
Influence of *I_min_* on the network convergence time in the small, medium and large network scenarios with different average node degrees as a function of the redundancy constant, *k*, when the *I_min_* value is set to 4 ms, 8 ms and 16 ms. (**a**) Small, (**b**) medium, and (**c**) large network size scenarios.

**Figure 11.**
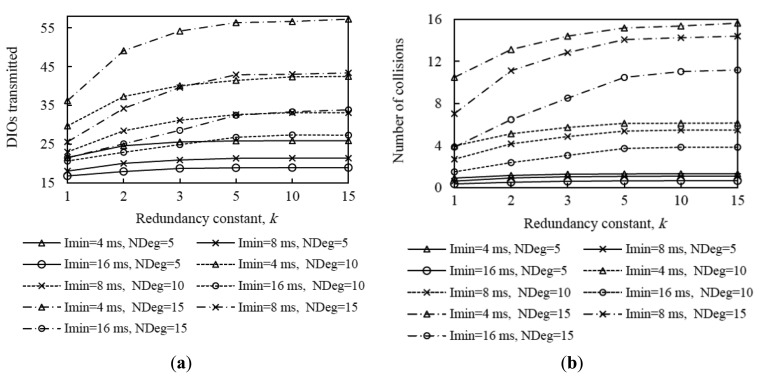
Average number of DIO messages transmitted and collisions in the small network scenario for different average node degrees, as a function of the redundancy constant, *k*, for different values of *I_min_*.

**Figure 12.**
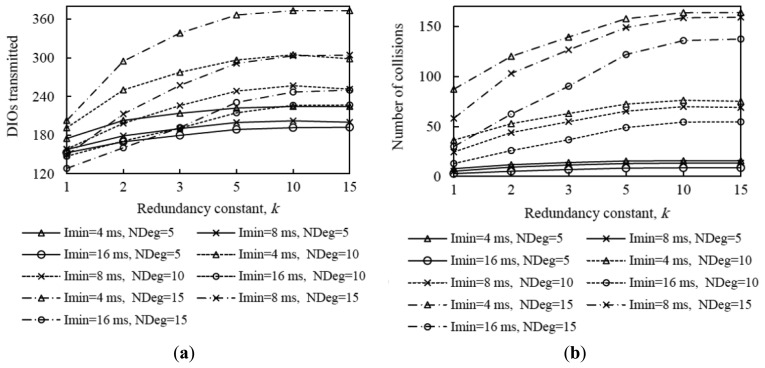
Average number of DIO messages transmitted and collisions in the medium network scenario for different average node degrees, as a function of the redundancy constant, *k*, for different values of *I_min_*.

**Figure 13.**
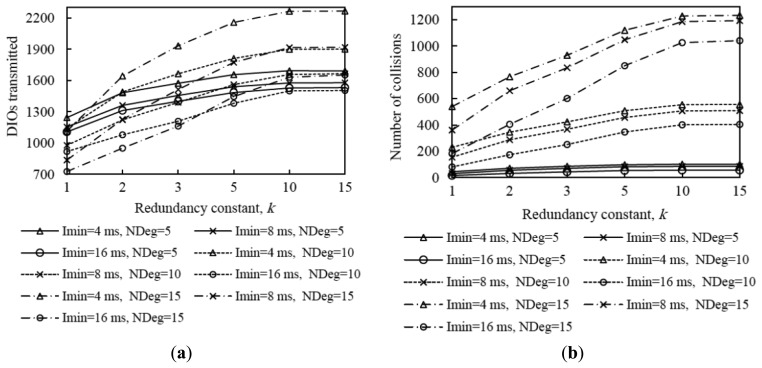
Average number of DIO messages transmitted and collisions in the large network scenario for different average node degrees, as a function of the redundancy constant, *k*, for different values of *I_min_*.

Figure 10 shows the average network convergence time when *I_min_* is set to 4 ms, 8 ms and 16 ms in the scenarios and for the settings presented in Section 4. As it can be seen in Figure 10, decreasing the minimum interval yields a reduction of network convergence time regardless of the network size, the network density or the redundancy constant, for the range of *I_min_* values considered. The reason is that when *I_min_* decreases, the length of Trickle intervals decreases as well, therefore nodes send their DIO messages earlier and more frequently, and as result a DODAG forms faster. Another important consideration that can be inferred from Figure 10 is that halving the *I_min_* value decreases the network convergence time by a factor smaller than two. As we already mentioned, when *I_min_* decreases, the number of DIO message transmissions during network convergence increases; as a consequence, the number of collisions increases as well (see Figures 11, 12 and 13). The number of collisions increase does not avoid the network convergence time decrease by decreasing *I_min_*, but it is the reason why this decrease is smaller than the *I_min_* decrease.

Analyzing in deeper detail the number of DIO messages transmitted during network convergence (Figures 11a, 12a and 13a), we observe that as both the redundancy constant and the network density grow, the influence of *I_min_* becomes greater. This is due to a lower amount of DIO message suppressions in the network. As a consequence of a greater number of DIO message transmissions, the number of collisions also grows as *I_min_* decreases (see Figures 11b, 12b and 13b). The relative influence of *I_min_* on the number of collisions does not vary with the redundancy constant, except for a high network density, whereby as the redundancy constant grows, the influence of *I_min_* becomes smaller. This happens because for a large *I_min_* value (e.g., *I_min_* = 16 ms), the number of collisions is low for low redundancy constant values. However, as the redundancy constant grows, and when the network is dense, the probability of collision significantly increases due to the greater number of DIO message transmissions in the network. On the contrary, for low *I_min_* values (e.g., *I_min_* = 4 ms), the number of collisions is already high for low redundancy constant values, since such *I_min_* values approach the DIO transmission time in the 2.4 GHz IEEE 802.15.4 physical layer (*i.e.*, 2.82 ms), and thus the number of collisions increase with the redundancy constant is smaller than that observed for greater *I_min_* values.

From the study carried out in this subsection, it can be derived that there exists a tradeoff between network convergence time and DIO message overhead (and collisions) that depends on *I_min_*. Varying *I_min_* in the range of values considered in this study has a greater quantitative effect on the number of DIO messages sent than on the network convergence time. Fine-tuning *I_min_* has a greater impact on the number of DIO messages transmitted during network convergence as both the redundancy constant and network density grow, whereas the relative impact of *I_min_* on network convergence time does not vary significantly with the redundancy constant, network size or network density.

## 6. DIS-Trickle: A Mechanism for Accelerating Network Convergence

This section proposes and evaluates a mechanism for leveraging DIS messages in order to accelerate RPL network convergence. In some scenarios a node may have to wait for a long time to receive a DIO message (and thus, to join a DODAG). As we described in Section 3.1, a node may discover nearby DODAG nodes in a short time by sending DIS messages, which will trigger a quick response (in the form of a DIO message) from neighbors that have already joined a DODAG. However, the RPL specification does not state any rule or scheduling algorithm for the transmission of DIS messages [5]. In this section we propose and evaluate DIS-Trickle, a mechanism for accelerating the network convergence process by using DIS messages. We first present DIS-Trickle and then evaluate its influence on network convergence performance.

### 6.1. DIS-Trickle Design

DIS-Trickle comprises two components: an initial delay and a scheduling algorithm. This subsection describes in detail both components.

For nodes that may send DIS messages, it is beneficial to introduce an initial delay before the first DIS message transmission. This is motivated by the fact that DIO messages need time to propagate through the network, and thus nodes at a multihop distance from the root node must have the opportunity of receiving DIO messages before sending premature, and unnecessary, DIS messages. The value of the initial delay has to be set carefully on the basis of the network size and density. Such an initial delay is also used in the COOJA/ContikiRPL implementation, whereby it is set by default to 5 s [28]. However, 5 s may be too large a value for the characteristics of many networks, including the ones considered in this paper. Based on simulation results that are discussed later in Section 6.2, we set the initial delay to 200 ms, which yields good performance in different network size and density scenarios, in terms of network convergence time, number of DIS and DIO messages transmitted, and collisions.

After the initial delay, in network zones where nodes have not yet received any DIO message, these nodes may attempt to transmit DIS messages at the same time, which may yield an *initial DIS storm* and a high number of collisions. On the other hand, it is not necessary that all non-DODAG neighbors of a DODAG node send a DIS message to trigger a DIO message transmission from the latter, since all these neighbors have the opportunity of receiving a DIO message sent by the DODAG node. In order to solve the *initial DIS storm* problem, as well as to limit unnecessary DIS message transmissions, we propose applying the Trickle algorithm to schedule the transmission of DIS messages. Since the main purpose of using DIS messages is the decrease of nodes' DODAG join time, we disable the Trickle interval size doubling mechanism in DIS-Trickle. On the other hand, in order to decrease the number of DIS message transmissions and the probability of message collisions while sending DIS messages, we set the redundancy constant of DIS-Trickle, *k_DIS_*, to 1. Another important parameter of DIS-Trickle is the interval size, denoted *I_DIS_*, which defines the time between consecutive DIS message transmissions. The value of this parameter should be specified on the basis of the DIS response time, defined as the time between the transmission of a DIS message and the corresponding DIO reply from a DODAG member. On the other hand, the time between consecutive DIS messages should not be unnecessarily large.

**Figure 14.**
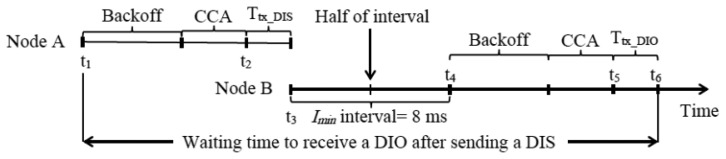
Calculation of the DIS response time. The figure illustrates the maximum delay between the transmission of a DIS message and the reception of a DIO message in response, where node A is interested in joining a DODAG and node B is already a DODAG member.

In order to assist the determination of *I_DIS_*, we next calculate the minimum and maximum DIS response time, assuming that nodes use a 2.4 GHz band IEEE 802.15.4 interface in the beaconles mode (see Figure 14). Let node A, which is interested in joining a DODAG, start the procedure for transmitting a DIS message at time *t_1_*. Let node B be already a DODAG member. The physical transmission of the DIS message starts after the backoff period and the CCA. We should note that, by default, the minimum and maximum backoff times in IEEE 802.15.4 are 0 ms and 16.96 ms, respectively. On the other hand, the time needed for performing the CCA on Telos B nodes running TinyOS is 3 ms. The physical layer of node A starts to send the DIS message at time *t_2_*, and it takes 1.34 ms (denoted by *T_tx_DIS_* in Figure 14) to transmit this message. On the other hand, node B, which has started to receive this DIS message at time *t_2_*, finishes receiving the DIS message at time *t_3_*, it immediately resets its Trickle timer to *I_min_* (which is set to 8 ms by default) and schedules itself to transmit a DIO message in the second half of the Trickle interval (of size *I_min_*). A DIO message transmission can take place at any point of the second half of the current interval, *i.e.*, it can suffer a random delay between 4 ms and 8 ms (the latter is illustrated in Figure 14). Finally, the physical transmission of this DIO message (which takes 2.82 ms) starts, after the backoff period and the CCA, at time *t_5_*, and node A finishes receiving the DIO message at time *t_6_*. Considering the duration of all the periods included between *t_1_* and *t_6_*, the DIS response time is a value between 14.16 and 52.08 ms. From the basis of this analysis, we tested different values for *I_DIS_*, (see the results obtained and discussed later in Section 6.2), and based on these tests we finally set *I_DIS_* to 30 ms, since it yields good performance in terms of both network convergence time and number of DIO and DIS messages transmitted during network convergence. Table 3 shows the default values of the DIS-Trickle parameters used in our simulations.

### 6.2. DIS-Trickle Evaluation

In this subsection, we evaluate DIS-Trickle and discuss the results obtained. We assume the default value of *I_min_* = 8 ms, analyze the influence of the redundancy constant on network convergence when DIS-Trickle is used, and consider the same range of network density and size scenarios evaluated in Section 5. We analyze the network convergence time, total number of RPL (*i.e.*, DIO and DIS) messages transmitted, and collisions during network convergence. We also study the impact of DIS-Trickle parameters on performance.

**Figure 15.**
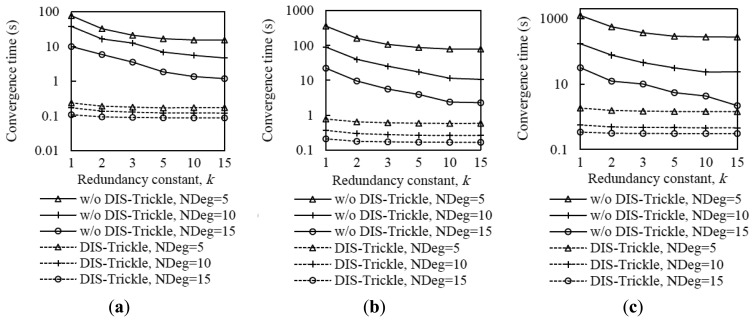
Influence of using DIS-Trickle on network convergence time in the small, medium and large network scenarios, for different average node degrees, NDeg, as a function of the redundancy constant, *k*, when the *I_min_* value is set to 8 ms. (**a**) Small network size, (**b**) medium network size, and (**c**) large network size.

As can be seen in Figure 15, using DIS-Trickle decreases network convergence time by 2 to 3 orders of magnitude, regardless of the network density and size. Actively requesting DIO messages constitutes a dramatically better strategy for non-DODAG nodes than passively waiting for DIO message reception, in terms of joining quickly a DODAG.

**Figure 16.**
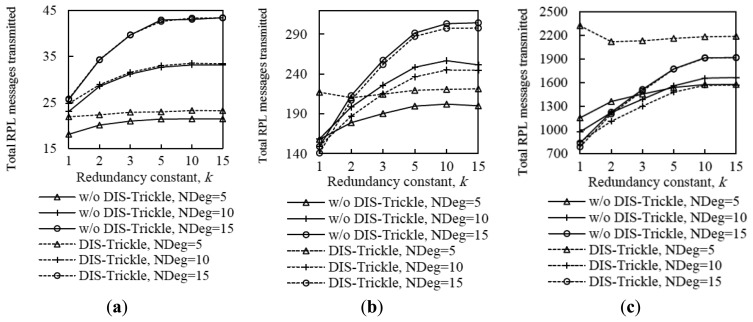
Influence of using DIS-Trickle on the total number of RPL messages (*i.e.*, DIO and DIS messages) transmitted in the small, medium and large networks, for different average node degrees as a function of the redundancy constant, *k*, when the *I_min_* value is set to 8 ms. (**a**) Small network size, (**b**) medium network size, and (**c**) large network size.

Interestingly, the network convergence time improvement yielded by DIS-Trickle does not always lead to performance degradation in terms of the total number of RPL (*i.e.*, DIO and DIS) messages sent. In dense networks, the RPL message overhead during network convergence does not grow, and even decreases, when DIS-Trickle is used (see Figure 16).

However, in sparse networks (e.g., when the average node degree is 5), DIS-Trickle requires a greater amount of total RPL message transmissions than that needed in absence of DIS-Trickle; impact of this phenomenon increases with network size. For a deeper analysis of the number of RPL messages sent during network convergence, we next study the number of DIO and DIS messages sent, separately.

**Figure 17.**
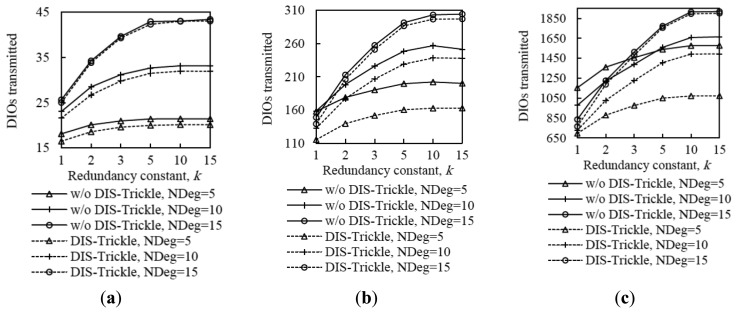
Influence of using DIS-Trickle on the number of DIO messages transmitted in the small, medium and large network scenarios, for different average node degrees, NDeg, as a function of the redundancy constant, *k*, when the *I_min_* value is set to 8 ms: (**a**) Small network size, (**b**) medium network size, and (**c**) large network size.

**Figure 18.**
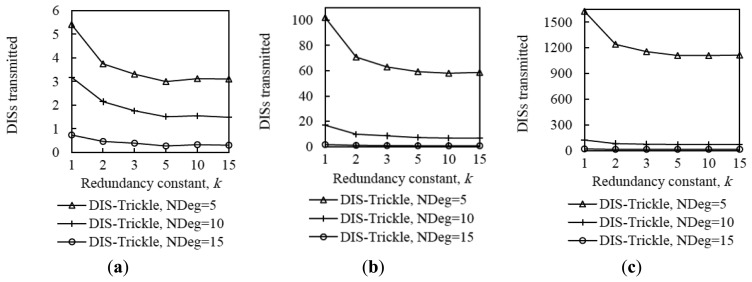
Influence of using DIS-Trickle on the number of DIS messages transmitted in the small, medium and large networks, for different average node degrees as a function of the redundancy constant, *k*. when the *I_min_* value is set to 8 ms. (**a**) Small network size, (**b**) medium network size, and (**c**) large network size.

Figure 17 shows that the average number of DIO messages transmitted decreases when DIS-Trickle is used. This reduction is more significant as network density decreases. The reason is that, as shown in subsection 5.1, in a sparse network, when DIS-Trickle is not used, the network convergence time is very high, and during this time a high number of DIO messages are transmitted until a DODAG is formed. However, using DIS-Trickle allows non-DODAG nodes to join a DODAG in a shorter time, and thus fewer DIO messages are required to complete the DODAG construction. On the other hand, when DIS-Trickle is used, the number of DIS message transmissions decreases as the redundancy constant grows (see Figure 18). This happens because as *k* increases, a greater number of DODAG nodes are able to send their DIO messages, therefore non-DODAG nodes have more chances to receive DIO messages, making it unnecessary to send DIS messages. Another important result is that the number of DIS message transmissions increases as network density decreases, since a lower amount of DIO messages are transmitted naturally by DODAG members. However, a large network size exacerbates the number of DIS message transmissions in sparse networks, due to the multiplicative effect of a greater number of end-to-end hops from the root to the most distant nodes. Therefore, in sparse networks, the high number of DIS message transmissions dominates the total number of RPL messages transmitted during network convergence, shown in Figure 16.

**Figure 19.**
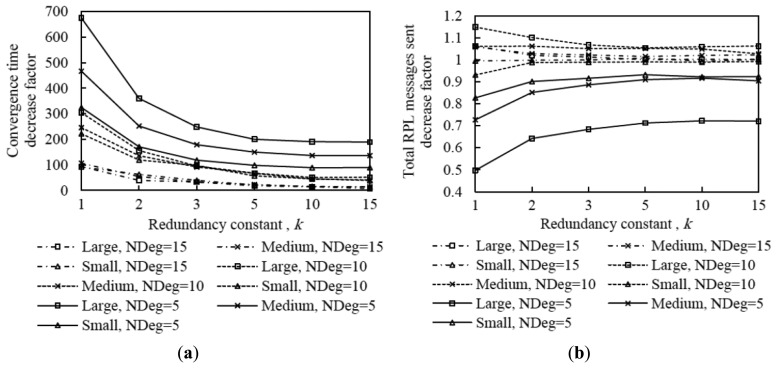
Convergence time (**a**) and number of DIO messages sent (**b**) improvement by using DIS-Trickle in three network size scenarios, for different average node degrees as a function of the redundancy constant, *k*.

Figure 19 illustrates the influence of network density, network size, and the redundancy constant setting on the relative performance of DIS-Trickle (*i.e.*, compared with not using DIS messages) in terms of both network convergence time and number of RPL message transmissions. In sparse networks (e.g., when the average node degree is 5), using DIS-Trickle improves network convergence time, but degrades the total number of RPL messages transmitted, since a high number of DIS message transmissions are required, as aforementioned.

As the network density increases, the network convergence time improvement due to using DIS-Trickle becomes lower, and does not vary significantly with the network size. This occurs because the probability that a node receives DIO messages without the need to send DIS messages increases with the network density. However, in dense networks, the total number of RPL message transmissions does not grow or even slightly decreases when DIS-Trickle is used. The reason for this result is that, in dense networks, a low number of DIS messages are required, and the DIS messages transmitted help reduce to a similar or even greater extent the number of DIO messages sent. Therefore, in dense networks, DIS-Trickle improves both network convergence time and number of RPL message transmissions, or at least does not degrade this last performance parameter (note that the result obtained in small networks, for a node degree of 10 and *k* = 1 is an exception to this conclusion, whereby the number of RPL message transmissions is slightly degraded when DIS-Trickle is used). Lastly, it can also be observed in Figure 19 that the influence of DIS-Trickle decreases as the redundancy constant grows, since a greater amount of DIO messages are naturally transmitted and a lower amount of DIS message transmissions are required.

**Figure 20.**
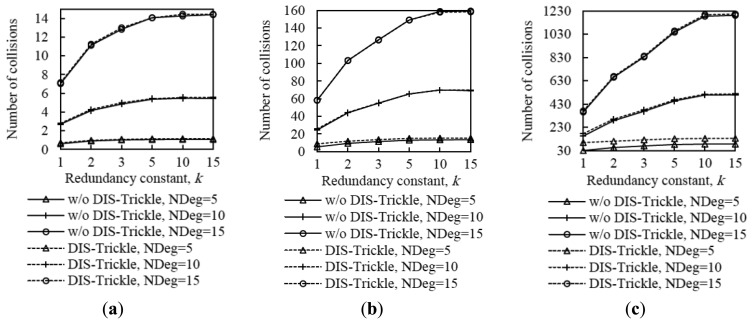
Influence of using DIS-Trickle on the number of collisions in the small, medium and large networks for different average node degrees, NDeg, as a function of the redundancy constant, *k*, when the *I_min_* value is set to 8 ms compared with not using DIS messages: (**a**) Small network size, (**b**) medium network size, and (**c**) large network size.

With regard to the number of collisions, for both DIO and DIS messages, it does not vary significantly by using DIS-Trickle (see Figure 20). This occurs because DIS messages are likely to be sent from nodes that have a low number of neighbors, and in addition, DIS-Trickle randomizes DIS message transmission time and suppresses redundant DIS message transmissions. Therefore, when DIS-Trickle is used, the number of message collisions does not significantly increase compared with the one observed in absence of DIS messages.

**Figure 21.**
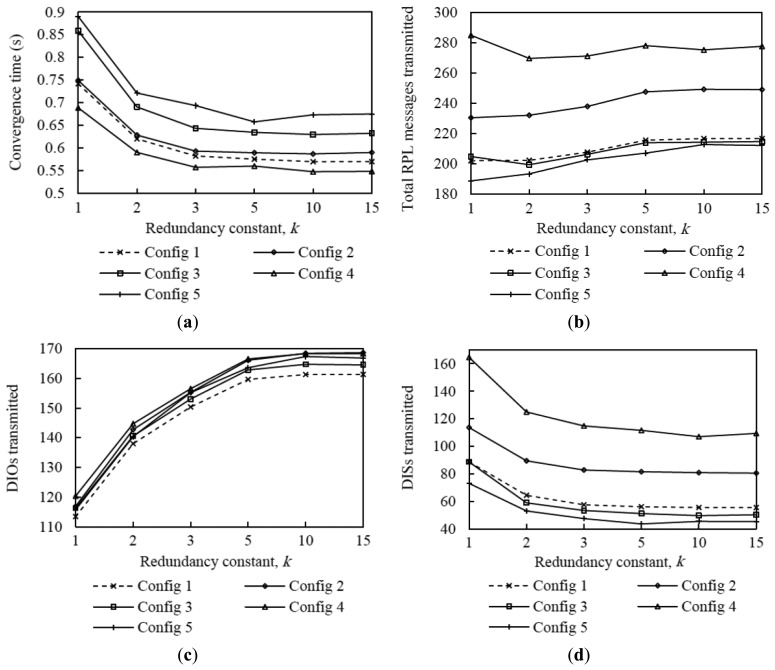

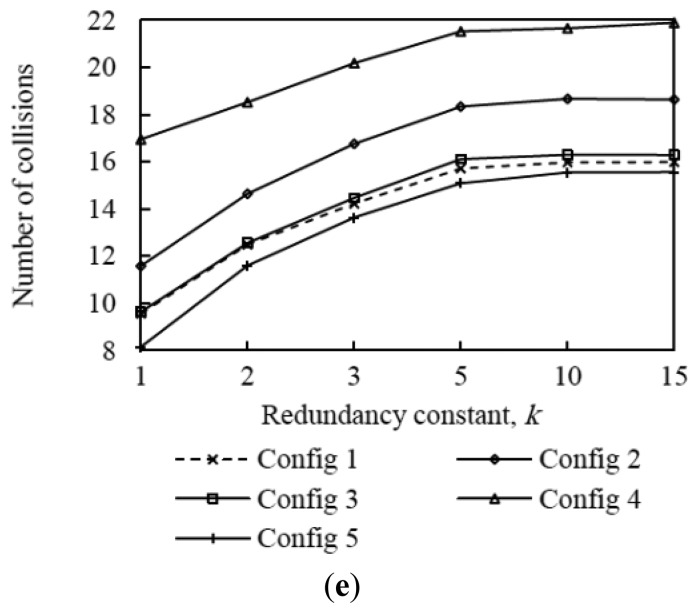
Comparing performance of different DIS-Trickle parameters settings (shown in Table 4) in medium network scenario when the average nodes degree is 5.

Finally, in order to understand the influence of *I_DIS_* and the DIS initial delay on performance of DIS-Trickle, we carried out simulations with different configurations of these DIS-Trickle parameters. The considered configurations of the aforementioned parameters are shown in Table 4. The evaluation is done in the medium network size scenario, for a node degree of 5. Note that this density corresponds to a sparse network and, as shown in Figure 4, the average network convergence time is high in the absence of the DIS-based mechanism, therefore we can observe the maximum influence of DIS-Trickle on network convergence performance within the range of considered network densities. The results obtained for these different configurations are plotted in Figure 21, which shows average results for the following performance parameters: network convergence time, number of DIO and DIS messages transmitted, and number of collisions (considering both DIO and DIS messages) during network convergence. Note that Configuration 1 (dashed line curve results in Figure 21) is the default set of DIS-Trickle parameter values used in our previous simulations.

As shown in Figures 21a and 21b, when the initial delay is set to 100 ms (Configuration 2), the network convergence time and the total number of RPL messages transmitted are greater than those obtained for an initial delay of 200 ms. The main reason is that 100 ms is lower than the time needed by DIO messages to propagate through the network. Thus, nodes that are not close to the root may start to prematurely (and unnecessarily) transmit DIS messages while their neighbors are receiving or sending DIO messages, increasing the number of collisions during the network convergence process (Figure 21e). On the other hand, when the initial delay is set to 300 ms (Configuration 3), network convergence time increases compared with using Configuration 2, due to the slower rate of DIS message generation (Figure 21d), while the rest of performance parameters do not vary significantly. As previously mentioned, a suitable value for the initial delay has to be set taking into account network characteristics such as density and size. From the results obtained, an initial delay of 200 ms offers good performance in the scenario considered, since it offers reasonably low network convergence time, low number of DIO and DIS message transmissions and low number of collisions.

Regarding the length of the DIS-Trickle interval, *I_DIS_*, we have tested values within the range of the DIS response time values presented in subsection 6.1. Figure 21 illustrates that, although a small value for *I_DIS_* such as 15 ms (Configuration 4), reduces slightly the network convergence time compared with the default *I_DIS_* value of 30 ms (Configuration 1), it causes the rest of performance parameters analyzed to significantly degrade. When *I_DIS_* is set to a low value such as 15 ms, many premature DIS message transmissions take place, leading also to a high number of collisions. On the other hand, using a greater value for *I_DIS_*, such as 45 ms (Configuration 5), has the opposite effect on performance. Results show that there is a tradeoff between the network convergence time and the rest of performance parameters that depends on *I_DIS_*. A medium *I_DIS_* value within the DIS response time range yields a good performance tradeoff.

## 7. Related Work

Despite the novelty of RPL, it has already been a subject of study in the literature [10–21]. However, only a few research works focus on or consider RPL network convergence. This section reviews related work, highlighting the differences of such work with the contributions and findings from this paper.

Authors of [12] present a simulation study of RPL based on the COOJA/ContikiRPL simulator. The main goal of this work is to investigate the impact of network parameters including number of nodes, number of DODAG roots, and packet loss on the performance of the network convergence process. The authors evaluate a range of network size values. Two different network topologies are considered: regular topologies, whereby nodes are quasi-uniformly distributed, and random topologies. A single value is tested for *I_min_*, which was set to 1 s. On the other hand, the redundancy constant value is not specified in the paper. Periodical transmission of DIS messages is evaluated in this work, based on a mechanism used in the COOJA/ContikiRPL [28] simulator. An initial delay is applied before the first DIS message transmission. The values used for the initial delay and for the length of the periodic intervals are 5 s and 60 s, respectively.

A survey about the most relevant RPL research efforts, along with an experimental performance evaluation of RPL, is presented in [13]. The performance evaluation of RPL provided by the authors includes network convergence time as a performance parameter, based on a single network size of 30 TelosB motes running ContikiRPL [28]. *I_min_* is set to a single value, equal to 4.096 s, which is the default value used in ContikiRPL. However, authors neither indicate which is the value of the redundancy constant nor whether DIS messages are used or not.

Another performance analysis of RPL that is also based on the COOJA/ContikiRPL simulator is presented in [21]. The authors evaluate control message overhead, network throughput and end-to-end packet delay in RPL for fixed values of *I_min_* and *k* set to 4.1 s and 10, respectively. This work considers a single area size and two different numbers of nodes: 20 and 100 nodes. However, the authors do not study the network convergence time. DIS messages are used in this work, but the parameter settings and further details on DIS message scheduling are not given.

A simulation-based performance evaluation of RPL for a single, medium size network is carried out by Tripathi *et al.* in [16]. Results include that global repair (*i.e.*, a mechanism that allows the root node to form a new DODAG when bad performance is detected) has a significant effect on the control message overhead. However, network convergence time is not studied in this work. *I_min_* is set to a single value (equal to 1 s), but the redundancy constant value used is not indicated. DIS-based mechanisms are not used in this work.

Clausen *et al.* investigate the efficiency of broadcast mechanisms in WSNs using RPL in [10,17]. The authors use two mechanisms for DIO transmission: (i) the Trickle algorithm, in which *I_min_* is set to a single value (equal to 2 s), and (ii) periodic DIO transmission. In both schemes the redundancy constant is set to infinity, *i.e.*, the suppression mechanism is disabled. The authors evaluate the network convergence time and message overhead parameters, based on a fixed node density (50 nodes/km^2^), for different network sizes in [10]. Results show that convergence time grows logarithmically with the number of nodes in the network. DIS-based mechanisms are not considered in the paper.

An experimental evaluation of RPL in TinyOS devices is provided in [11]. Regarding RPL parameters, a single value of *I_min_* is considered (*I_min_* = 128 ms), whereas the paper does not mention the redundancy constant setting. In order to study the impact of the *I_min_* value on the network convergence time, the authors present experimental in a single size and single density network of 40 nodes, with perfect and also with lossy channels for three different values of *I_min_*: 0.25 s, 1 s and 4 s. Results show that network convergence time increases with *I_min_*, which is consistent with results obtained in this paper. However, the influence of *I_min_* on other performance parameters such as RPL message overhead or collisions, has not been considered in the work. DIS messages have been included in the work, however details about DIS message scheduling rules or related parameters are not provided by the authors.

Analytical models have also been developed to estimate the network convergence time in a Trickle-based network. Authors in [14] provide such a model for line and grid network scenarios, based on a method to derive the probability density function of the time until network consistency through the use of Laplace transforms. However, the complexity of the analysis increases very quickly with the network diameter. Furthermore, the models presented do not take into account physical and link layer characteristics. Other network convergence time analytical models for Trickle-based networks are provided for a single-hop, and for a grid network scenario in [15]. These models neither take into account radio interface characteristics, and assume error-free networks.

Despite the fact that RPL has already been evaluated from different perspectives in the literature, to the best of our knowledge there is not any published research study that provides comprehensive insight on the influence of RPL parameters and mechanisms, and network characteristics, on the network convergence process in RPL.

## 8. Conclusions

In this paper we investigated by extensive simulation the influence of two important RPL parameters, the redundancy constant, *k*, and the minimum interval, *I_min_*, on the network convergence process, on top of a variety of IEEE 802.15.4 multihop network densities and sizes. We also proposed and evaluated a mechanism called DIS-Trickle for accelerating the network convergence process by exploiting DIS messages.

Results show that using low values for the redundancy constant yields high network convergence time in all network size and density scenarios considered, since a high number of DIO messages are suppressed by the Trickle algorithm. As the redundancy constant increases up to medium values, the network convergence time decreases. However, network convergence times do not vary significantly as the value of *k* increases beyond the values around the node degree. There exists a tradeoff between network convergence time and other performance parameters, such as the number of DIO messages transmitted and number of collisions that depends on *k*. In order to achieve a low network convergence time, the redundancy constant should be set to a value equal or close to the average node degree, since greater values might cause an unnecessary increase in the number of DIO message transmissions and collisions in very dense network areas, which however would not yield a network convergence time decrease. As network density increases, the influence of the redundancy constant grows with the network size. In sparse networks, the relative influence of *k* is independent of the network size.

With regard to the minimum interval, we found that there exists a tradeoff between network convergence time and DIO message overhead (and collisions) that depends on *I_min_*. Varying *I_min_* in the range of values considered in this study has a greater quantitative effect on the number of DIO messages sent than on the network convergence time. Fine-tuning *I_min_* has a greater impact on the number of DIO messages transmitted during network convergence as both the redundancy constant and network density grow, whereas the impact of *I_min_* on network convergence time does not vary significantly with the redundancy constant, network size or network density.

Finally, we proposed and evaluated DIS-Trickle, a mechanism that leverages DIS messages in order to reduce nodes' DODAG join time. Results show that using DIS-Trickle decreases network convergence time in 2–3 orders of magnitude, regardless of the network size or density. The improvement provided by this mechanism grows as network density decreases. Interestingly, in dense networks, DIS-Trickle does not increase or even reduces the total number of RPL messages sent during network convergence. However, in sparse networks, DIS-Trickle creates a trade-off between network convergence time and RPL message overhead. The influence of DIS-Trickle on network convergence performance decreases with the redundancy constant.

The findings and contributions in this work offer a guideline for configuring and selecting adequately RPL parameters and mechanisms for achieving a good network convergence performance tradeoff, on the basis of network characteristics such as size and density.

## Figures and Tables

**Table 1.** Main physical and link layer simulation parameters.

**Parameter**	**Value**
Communication range (m)	9.96
Carrier frequency (GHz)	2.4
Carrier sense sensitivity (dBm)	−95
Transmit power (dBm)	−25
MAC queue length	1

**Table 2.** Number of nodes and average rank for each network size scenario and density.

**Network Size**	**Node Degree**	**Number of Nodes**	**Average Rank**
Small	5	8	3.09
	10	14	3.30
	15	21	3.30

Medium	5	34	6.52
	10	66	6.34
	15	99	5.78

Large	5	162	16.76
	10	322	12.43
	15	483	10.74

**Table 3.** DIS-Trickle parameter configuration.

**Parameter**	**Value**
Initial delay (ms)	200
Interval length, *I_DIS_* (ms)	30
Number of doublings	0
DIS-Trickle redundancy constant, *k_DIS_*	1

**Table 4.** DIS-Trickle parameter configurations evaluated.

**Parameter**	**Configuration**				

	**1**	**2**	**3**	**4**	**5**
DIS initial delay	200 ms	100 ms	300 ms	200 ms	200 ms
Interval length, *I_DIS_*	30 ms	30 ms	30 ms	15 ms	45 ms

## References

[1] Gomez C., Paradells J., Caballero J.E. (2010). Sensors Everywhere: Wireless Network Technologies and Solutions.

[2] Karl H., Willig A. (2005). Protocols and Architecture of Wireless Sensor Networks.

[3] Ishaq I., Carels D., Teklemariam G.K., Hoebeke J., Abeele F.V., Poorter E.D., Moerman I., Demeester P. (2013). IETF Standardization in the Field of the Internet of Things (IoT): A Survey. J. Sens. Actuator Net..

[4] ROLL Charter http://datatracker.ietf.org/wg/roll/charter/.

[5] Winter T., Thubert P., Brandt A., Hui J., Kelsey R., Levis P., Pister K., Struik R., Vasseur J.P., Alexander R. RPL: IPv6 Routing Protocol for Low power and Lossy Networks. RFC 6550.

[6] Brandt A., Buron J., Porcu G. Home Automation Routing Requirements in Low-Power and Lossy Networks. RFC 5826.

[7] Martocci J., De Mil P., Riou N., Vermeylen W. Building Automation Routing Requirements in Low-Power and Lossy Networks. RFC 5867.

[8] Pister K., Thubert P., Dwars S., Phinney T. Industrial Routing Requirements in Low-Power and Lossy Networks. RFC 5673.

[9] Dohler M., Watteyne T., Winter T., Barthel D. Routing Requirements for Urban Low-Power and Lossy Networks. RFC 5548.

[10] Clausen T., Herberg U. (2010). Multipoint-to-Point and Broadcast in RPL..

[11] Ko J., Dawson-Haggerty S., Gnawali O., Culler D., Terzis A. Evaluating the performance of RPL and 6LoWPAN in TinyOS.

[12] Gaddour O., Koub A., Chaudhry S., Tezeghdanti M., Chaari R., Abid M. Simulation and Performance Evaluation of DAG Construction with RPL.

[13] Gaddour O., KoubíA A. (2012). RPL in a nutshell: A survey. Comput. Netw..

[14] Becker M., Kuladinithi K., Görg C. Modelling and simulating the Trickle algorithm.

[15] Meyfroyt T.M.M. (2013). Modeling and Analyzing the Trickle Algorithm. Master's Thesis.

[16] Tripathi A., de Oliveira J., Vasseur J. Performance Evaluation of Routing Protocol for Low Power and Lossy Networks. RFC 6687.

[17] Clausen T., Herberg U. Comparative study of RPL-enabled optimized broadcast in wireless sensor networks.

[18] Radoi I.E., Shenoy A., Arvind D.K. Evaluation of Routing Protocols for Internet-Enabled Wireless Sensor Networks.

[19] Ben Saad L., Chauvenet C., Tourancheau B. Simulation of the RPL Routing Protocol for IPv6 Sensor Networks: Two Cases Studies.

[20] Kermajani H., Gomez C., Arshad M.H. (2012). Modeling the message count of the Trickle algorithm in a steady-state, static wireless sensor network. IEEE Commun. Lett..

[21] Accettura N., Grieco L.A., Boggia G., Camarda P. Performance Analysis of the RPL Routing Protocol.

[22] IEEE Std. 802.15.4–2006 IEEE Standard for Information Technology—Telecommunications and Information Exchange between Systems—Local and Metropolitan Area Networks—Specic Requirements Part 15.4: Wireless Medium Access Control (MAC) and Physical Layer (PHY) Specifications for Low-Rate Wireless Personal Area Networks (WPANs). http://ieeexplore.ieee.org/xpl/articleDetails.jsp?tp=&arnumber=4040999&url=http%3A%2F%;2Fieeexplore.ieee.org%2FielD%2F4040997%2F4040998%2F04040999.

[23] Clausen T., Yi J., Herberg U. Experieces with RPL: IPv6 Routing Protocol for Low power and Lossy Networks.

[24] Xie W., Goyal M., Mosseini H., Martocci J., Bashir Y., Baccelli E., Durresi A. Routing Loops in DAG-based Low Power and Lossy Networks.

[25] Levis P., Clausen T., Hui J., Gnawali O., Ko J. The Trickle Algorithm. RFC 6206.

[26] Thubert P. RPL. Objective Function Zero. RFC 6552.

[27] Goyal M., Baccelli E., Philipp M., Brandt A., Martocci J. Reactive Discovery of Point-to-Point Routes in Low-Power and Lossy Networks. RFC 6997.

[28] COOJA/ContikiRPL simulator. http://www.contiki-os.org/start.html.

[29] Varga A. The omnet++ discrete event simulation systems.

[30] MiXiM simulator for wireless and mobile networks using OMNeT++. http://mixim.sourceforge.net/.

[31] Kermajani H. RPL. simulation code for OMNeT++. https://sites.google.com/site/carlesgomez/home/code.

[32] Texas Instruments Incorporated 2.4 GHz IEEE 802.15.4/ZigBee-ready RF Transceiver. http://www.ti.com/lit/gpn/cc2420.

[33] TELOSB MOTE PLATFORM http://www.willow.co.uk/TelosB_Datasheet.pdf.

[34] Levis P., Madden S., Polastre J., Szewczyk R., Whitehouse K., Woo A., Gay D., Hill J., Welsh M., Brewer E. (2005). TinyOS: An Operating System for Sensor Networks. Ambient Intelligence.

